# Chronic Exposure to Chewing Tobacco Induces Metabolic Reprogramming and Cancer Stem Cell-Like Properties in Esophageal Epithelial Cells

**DOI:** 10.3390/cells8090949

**Published:** 2019-08-21

**Authors:** Keshava K. Datta, Shankargouda Patil, Krishna Patel, Niraj Babu, Remya Raja, Vishalakshi Nanjappa, Kiran Kumar Mangalaparthi, Bharti Dhaka, Pavithra Rajagopalan, Sayali Chandrashekhar Deolankar, Ramakrishnan Kannan, Prashant Kumar, T. S. Keshava Prasad, Premendu P. Mathur, Anjali Kumari, Malini Manoharan, Karunakaran Coral, Saktivel Murugan, David Sidransky, Ravi Gupta, Rohit Gupta, Arati Khanna-Gupta, Aditi Chatterjee, Harsha Gowda

**Affiliations:** 1 Institute of Bioinformatics, International Tech Park, Bangalore 560066, India; 2 Department of Maxillofacial Surgery and Diagnostic Sciences, Division of Oral Pathology, College of Dentistry, Jazan University, Jazan 45142, Saudi Arabia; 3 Department of Medical Biotechnologies, School of Dental Medicine, University of Siena, 53100 Siena, Italy; 4 Amrita School of Biotechnology, Amrita Vishwa Vidyapeetham, Kollam 690525, India; 5 Manipal Academy of Higher Education (MAHE), Madhav Nagar, Manipal 576104, India; 6 Center for Systems Biology and Molecular Medicine, Yenepoya (Deemed to be University), Mangalore 575018, India; 7 National Institute of Mental Health and Neuro Sciences (NIMHANS), Hosur Road, Bangalore 560029, India; 8 School of Biotechnology, KIIT (Deemed to be University), Bhubaneswar 751024, India; 9 Department of Biochemistry and Molecular Biology, School of Life Sciences, Pondicherry University, Pondicherry 605014, India; 10 Medgenome Labs Pvt. Ltd., Bangalore 560099, India; 11 Department of Otolaryngology-Head and Neck Surgery, Johns Hopkins University School of Medicine, Baltimore, MD 21231, USA

**Keywords:** mitochondria, cancer metabolism, tobacco, proteomics, exome sequencing, electron microscopy

## Abstract

Tobacco in its smoke and smokeless form are major risk factors for esophageal squamous cell carcinoma (ESCC). However, molecular alterations associated with smokeless tobacco exposure are poorly understood. In the Indian subcontinent, tobacco is predominantly consumed in chewing form. An understanding of molecular alterations associated with chewing tobacco exposure is vital for identifying molecular markers and potential targets. We developed an in vitro cellular model by exposing non-transformed esophageal epithelial cells to chewing tobacco over an eight-month period. Chronic exposure to chewing tobacco led to increase in cell proliferation, invasive ability and anchorage independent growth, indicating cell transformation. Molecular alterations associated with chewing tobacco exposure were characterized by carrying out exome sequencing and quantitative proteomic profiling of parental cells and chewing tobacco exposed cells. Quantitative proteomic analysis revealed increased expression of cancer stem cell markers in tobacco treated cells. In addition, tobacco exposed cells showed the Oxidative Phosphorylation (OXPHOS) phenotype with decreased expression of enzymes associated with glycolytic pathway and increased expression of a large number of mitochondrial proteins involved in electron transport chain as well as enzymes of the tricarboxylic acid (TCA) cycle. Electron micrographs revealed increase in number and size of mitochondria. Based on these observations, we propose that chronic exposure of esophageal epithelial cells to tobacco leads to cancer stem cell-like phenotype. These cells show the characteristic OXPHOS phenotype, which can be potentially targeted as a therapeutic strategy.

## 1. Introduction

Esophageal carcinoma (EC) is the eighth most common cancer affecting >450,000 people worldwide. India accounts for >10% of all EC cases. Approximately 400,000 deaths were recorded in 2012 due to EC, making it the sixth common cause of cancer deaths [1]. Based on histology, EC is classified into squamous cell carcinoma (ESCC) and adenocarcinoma (EAC). ESCC is the predominant subtype of EC worldwide [2]. Tobacco use, both in its smoking and smokeless forms, are well-known risk factors for cancer. Smokeless tobacco is tobacco that is not burnt, including chewing tobacco and snuff. Smokeless tobacco is a known risk factor for oral, esophageal and pancreatic cancer [3]. India is the largest consumer of chewing tobacco, accounting for 80% of global tobacco chewers [4]. Although case-control studies undertaken in different parts of India have demonstrated that tobacco chewing is a major risk factor for ESCC [5,6], molecular alterations associated with tobacco chewing have not been systematically investigated. A better understanding of molecular alterations associated with exposure to chewing tobacco will provide biomarkers that can be used for diagnosis and monitoring and identify novel targets for therapeutic intervention.

Neoplastic transformation of cells is a multistep process. Several molecular alterations that transform non-neoplastic cells have been characterized over the years. Most of these are genetic changes that occur in the genomic DNA due to carcinogens, spontaneous mutations or viral infections that integrate mutant oncogenes into genomic DNA [7,8]. These genomic alterations subsequently drive biochemical processes that promote cell proliferation. This includes aberrant activation of signaling pathways and metabolic reprogramming [9]. Under anaerobic conditions, normal cells favor glycolysis for energy production [10]. Cancer cells, however, derive their energy largely from glycolysis, even in the presence of oxygen [11]. This metabolic switch, termed “aerobic glycolysis” or “the Warburg effect”, facilitates the usage of glycolytic intermediates as precursors for organelles and macromolecules that are required for new cells [12]. A growing body of literature suggests that bulk cancer cells and cancer stem cells (CSCs) have different metabolic phenotypes. Studies conducted in lung cancer [13], pancreatic cancer [14], ovarian cancer [15] and acute myeloid leukemia [16], have demonstrated that CSCs prefer OXPHOS for energy production. 

Non-neoplastic cell lines serve as valuable models for investigating molecular alterations associated with chronic exposure to carcinogenic substances. These model systems have been extensively used to investigate effects of nicotine and tobacco smoke condensate [17]. In this study, we treated Het1A, a non-neoplastic esophageal cell line, with chewing tobacco extract over a period of eight months. Changes associated with these chewing tobacco exposed cells were monitored by microscopy and phenotypic assays including cell proliferation, invasion and colony formation. Cells that showed oncogenic transformation were then characterized by carrying out whole exome sequencing and global proteomic profiling.

## 2. Materials and Methods

### 2.1. Cell Culture and Tobacco Treatment

Het1A, a non-neoplastic and non-transformed epithelial cell line from the human esophagus was procured from American Type Culture Collection (ATCC, Manassas, VA, USA). It was cultured in keratinocyte serum free medium supplemented with 25 µg/mL bovine pituitary extract, 0.2 ng/mL epidermal growth factor, 1% penicillin/streptomycin and 0.4 mM CaCl_2_. Chewing tobacco extract was prepared as described earlier [18]. Cells were treated with chewing tobacco extract at a final concentration of 1%. The parental and treated cells were grown at 37 °C in a humidified 5% CO_2_ incubator. Henceforth, tobacco treated cells, based on the duration of treatment, will be termed as Het1A-2M, Het1A-4M, Het1A-6M and Het1A-8M. Parental cells that were not treated with tobacco were cultured in parallel for the same duration and will be referred to as Het1A-P cells.

### 2.2. Cell Culture-Based Assays

Proliferation assay: Het1A-P and treated cell lines (Het1A-2M, Het1A-4M, Het1A-6M and Het1A-8M) were seeded at a density of 50 × 10^3^ cells per well in 6-well plates. Cell proliferation was monitored for 6 days by counting cells every 48 h using trypan blue exclusion method.

Invasion assay: the invasive ability of Het1A-P and Het1A-8M was studied using a transwell system (BD Biosciences, San Jose, CA, USA) with Matrigel (BD Biosciences, San Jose, CA, USA) coated filters, as described previously [19]. Briefly, invasiveness of the cells was assayed in the membrane invasion culture system using polyethylene terephthalate (PET) membrane (8-μm pore size) in the upper compartment of a transwell coated with Matrigel (BD BioCoat Matrigel Invasion Chamber; BD Biosciences). Per well, 20,000 cells were seeded, along with 500 μL of media on the Matrigel-coated PET membrane in the upper compartment. The lower compartment was filled with complete growth media and the plates were maintained at 37 °C for 48 h. At the end of the incubation time, the upper surface of the membrane was wiped with a cotton-tip applicator to remove non migratory cells. Cells that migrated to bottom side of membrane were fixed and stained using 4% methylene blue. The number of cells that invaded was counted for 10 randomly selected viewing fields. 

Soft-agar assay: anchorage independent growth capability of Het1A-P and Het1A-8M was investigated as described earlier [20]. Briefly, 5 × 10^3^ cells were mixed with 1 mL of media containing 0.3% low-melting agarose and poured onto a bed of 1 mL per well media containing 0.5% agarose in a six well plate. Colonies were stained and photographed after 12 days.

All assays were performed in triplicates and repeated thrice.

### 2.3. Western Blotting

Apoptotic markers were probed using β-actin as loading control as described earlier [18].

### 2.4. SOD Assay

SOD determination kit was procured from Sigma Aldrich (St. Louis, MO, USA) and the assay was carried out as per the manufacturer’s protocol. Briefly, equal number of cells from Het1A-P and Het1A-8M were lysed in 0.1M Tris-HCl (pH 7.4), containing 0.5% Triton X, 0.1 mg/mL PMSF. The lysates were centrifuged at 14,000 *g* for 5 min and the supernatant was collected. 20 µL of these samples were mixed with 200 µL of WST working solution and 20 µL of enzyme working solution and incubated at 37 °C for 20 min. Absorbance was recorded at 450 nm and SOD activity was calculated. 

### 2.5. Sample Preparation for Exome Sequencing

DNA extracted from Het1A-P and Het1A-8M was sonicated to produce sheared fragments in the size range of 150–200 base pair length. DNA library for exome sequencing was prepared using Agilent SureSelectXT Human All Exon V5 kit as per manufacturer’s instructions. DNA fragments obtained from shearing were end-repaired and phosphorylated, followed by adenylation of 3’ ends and ligation of standard paired end adaptors. Hybridization was carried out at 65 °C for 16 h using DNA library with addition of biotin-labeled RNA probe sets, designed specifically for the desired targets. Dynabeads^®^ MyOne™ Streptavidin T1 beads were used for capture of resulting DNA-RNA duplexes. Multiple washes at high stringency were performed to remove any bound off-target material and any non-hybridized fragments. Specific libraries were then amplified using indexed primers and Herculase II Fusion DNA Polymerase (Agilent Technologies Inc., Santa Clara, CA, USA). Subsequently, cluster amplification was performed according to manufacturer’s protocol (Illumina Inc., San Diego, CA, USA) using V3 Chemistry and V3 flowcells. Paired-end sequencing was performed on Illumina HiSeq 2500 with read length of 100 bp for the whole exome using the TruSeq Cluster Kit v3.

### 2.6. Exome Data Analysis

Bases with Phred quality score < 20 were trimmed from the reads and reads shorter than 35 nt were excluded. Trimmed sequencing reads were then aligned to the human reference genome version hg19 (GRCh37) using BWA (Burrows-Wheeler Aligner)-MEM (Maximal Exact Matches) [21]. After alignment process, we employed Genome Analysis Toolkit (GATK) processing pipeline before calling somatic variations. These steps included the removal of duplicates using MarkDuplicates of Picard tools to minimize experimental artifacts, indel realignment using IndelRealigner and base recalibration using BaseRecalibrator of the GATK tool suite (the Genome Analysis Toolkit, Broad Institute). This approach has been widely used to improve variant calling accuracy.

Somatic single nucleotide variants and small indels were called using Strelka [22] within the target interval of exome capture kit. To identify high confidence variants, we applied the following post-processing filters: (1) loci with ≥10 reads in Het1A-8M and ≥8 in Het1A-P were used for variant calling. (2) Alternate alleles were supported by at least 15% of the total reads in Het1A-8M cells. Variant annotation was carried out with Varimat with in-house database OncoMD and publicly available databases such as 1000 Genome Project database, dbSNP147, COSMIC (Catalogue of Somatic Mutations in Cancer) and ICGC (International Cancer Genome Consortium). After annotation, single nucleotide variants (SNVs) were classified into several categories based on genomic functional regions and their potential functional impacts (non-frameshift and frameshift indels, non-synonymous and synonymous SNVs and stop-gain and stop-loss variants). Polyphen and SIFT were used to predict potential functional effect of non-synonymous single nucleotide variants. Finally, a list of candidate somatic mutations was generated that includes only non-synonymous SNVs filtered variants registered in dbSNP147 database with Minor Allele Frequency ≥ 0.05.

We employed OncoCNV [23] to identify copy number alterations (CNAs) by comparing Het1A-8M with Het1A-P to infer somatic CNAs. CNAs ≥ 3 was used as a threshold to call amplifications and ≤0.5 was used to call deletions.

### 2.7. Sample Preparation for Proteomic Analysis

Het1A-P and treated cell lines (Het1A-2M, Het1A-4M, Het1A-6M and Het1A-8M) were grown to ~80% confluence and washed with 1× PBS thrice and harvested in lysis buffer (2% SDS in 50 mM Triethyl Ammonium Bicarbonate with protease inhibitors). The cell lysates were sonicated, centrifuged and protein concentration was determined by bicinchoninic acid assay. Equal amount of protein samples from all conditions were subjected to reduction and alkylation using 5 mM dithiothreitol and 20 mM iodoacetamide, respectively. Proteins were precipitated using ice cold acetone and in-solution trypsin digestion of samples was carried out using 1:20 enzyme-to-substrate ratio. Trypsin digested peptides were lyophilized and labeled with Tandem Mass Tags (TMT) as per manufacturer’s protocol. TMT labeled peptides were pooled and subjected to basic pH reverse phase liquid chromatography (bRPLC)-based fractionation [24]. A total of 12 fractions were analyzed in triplicates on an Orbitrap Fusion Tribrid mass spectrometer (Thermo Electron, Bremen, Germany) as described earlier [25].

### 2.8. Proteomic Data Analysis

Raw files obtained from mass spectrometry analysis were searched against Human RefSeq protein database using Sequest HT and Mascot search algorithms through Proteome Discoverer (version 2.1) (Thermo Scientific, Bremen, Germany). Precursor and fragment mass tolerance were set to 10 ppm and 0.05 Da, respectively. TMT at N-terminus and lysine and the carbamidomethylation of cysteine were set as fixed modifications, while oxidation of methionine was set as dynamic modification. A false discovery rate (FDR) cut-off of 1% was used to filter peptide spectrum matches (PSMs). FDR was calculated using a decoy search.

Mass spectrometry data generated in this study has been deposited to the ProteomeXchange Consortium through the PRIDE partner repository with dataset identifier PXD013396.

### 2.9. Electron Microscopy

All chemicals and solvents were obtained from TAAB Laboratories Equipment Ltd., UK. Het1A-P and Het1A-8M cell pellets were fixed with 3% buffered glutaraldehyde followed by secondary fixation with 1% buffered osmium tetroxide. Fixed cell pellets were further processed for electron microscopy. Uranyl acetate and lead citrate staining was performed on ultra-thin sections and were observed under FEI Tecnai electron microscope.

### 2.10. Statistical Analysis

All statistical analyses were carried out using GraphPad Prism version 6 (GraphPad Software, La Jolla, CA, USA). For cell culture-based assays (proliferation, invasion and soft-agar), Western blots, and electron microscopy, non-parametric test (Mann-Whitney U test) was used to assess statistical significance between Het1A-P and Het1A-8M. For proteomics data, Tukey’s multiple comparison tests were used to assess statistical significance.

## 3. Results

Non-neoplastic esophageal epithelial cells (Het1A) were treated with various concentrations of chewing tobacco extract (0.1–5%) to determine optimum concentration for chronic treatment. Highest tobacco concentration at which we observed minimal cytotoxicity was at 1% (data not shown). Thus, 1% tobacco extract was used for chronic treatment of Het1A cells for a period of 8 months. 

### 3.1. Chronic Exposure to Tobacco Extract Leads to Cancer Cell Phenotype in Esophageal Epithelial Cells

**Figure 1:**
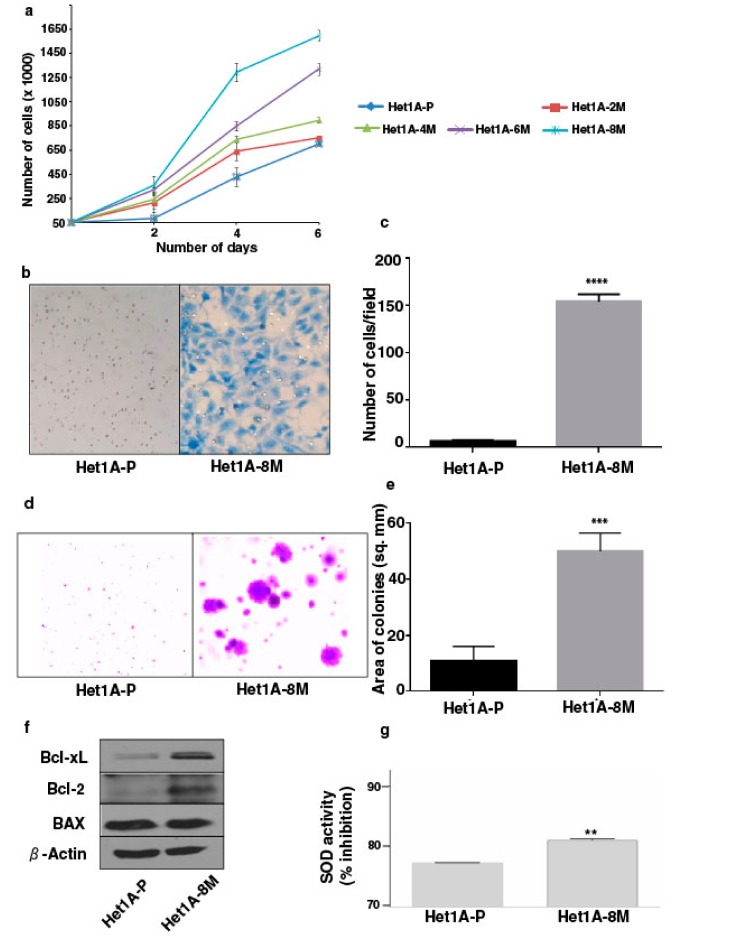
Chronic exposure to chewing tobacco induces oncogenic phenotype in esophageal epithelial cells. (**a**) Proliferation curves of parental and chewing tobacco exposed Het1A cells. (**b**) Representative image of cell invasion by parental and Het1A-8M cells (magnification = 100×). (**c**) Quantification of invasion capabilities of parental and Het1A-8M cells. (**d**) Representative image of anchorage independent growth by parental and Het1A-8M cells. (**e**) Quantification of anchorage independent growth of parental and Het1A-8M cells. (**f**) Immunoblot of Bcl-xL, Bcl-2, and BAX in parental and Het1A-8M cells. (**g**) Superoxide dismutase (SOD) activity in parental and Het1A-8M cells. ** (*p* ≤ 0.01), *** (*p* ≤ 0.001), **** (*p* ≤ 0.0001).

Increased cell proliferation and invasive capability are characteristic features of oncogenic transformation. We observed progressive increase in the proliferative ability of esophageal epithelial cells with increased duration of exposure to chewing tobacco (Figure 1a). Matrigel-based in vitro invasion assay showed that Het1A-8M cells gained significant invasive capability following chronic tobacco exposure (Figure 1b,c). It has been demonstrated that anchorage-independence correlates strongly with tumorigenicity [26]. We employed soft agar assay to evaluate anchorage independent growth capability of tobacco treated cells (Figure 1d). Het1A-8M cells formed significantly larger colonies compared to Het1A-P cells (Figure 1e), indicating potential oncogenic transformation.

Cancer cells often show altered expression of pro-apoptotic and anti-apoptotic proteins to escape cell death [27]. We evaluated expression levels of apoptotic proteins using western blots. Het1A-8M cells showed increased expression levels of both Bcl-xL and Bcl-2, while the expression level of BAX was unchanged (Figure 1f, Supplementary Materials Figure S3). Genotoxic effects caused by carcinogenic constituents of tobacco are known to raise the levels of reactive oxygen species (ROS) in epithelial cells [28]. Superoxide dismutases (SODs) are the major defense systems against excess ROS [29]. We measured SOD activity and found that it was significantly higher in Het1A-8M cells, implying that tobacco extract increased ROS levels in the cells (Figure 1g).

### 3.2. Genomic Alterations Associated with Esophageal Epithelial Cells Chronically Exposed to Tobacco Extract

**Figure 2:**
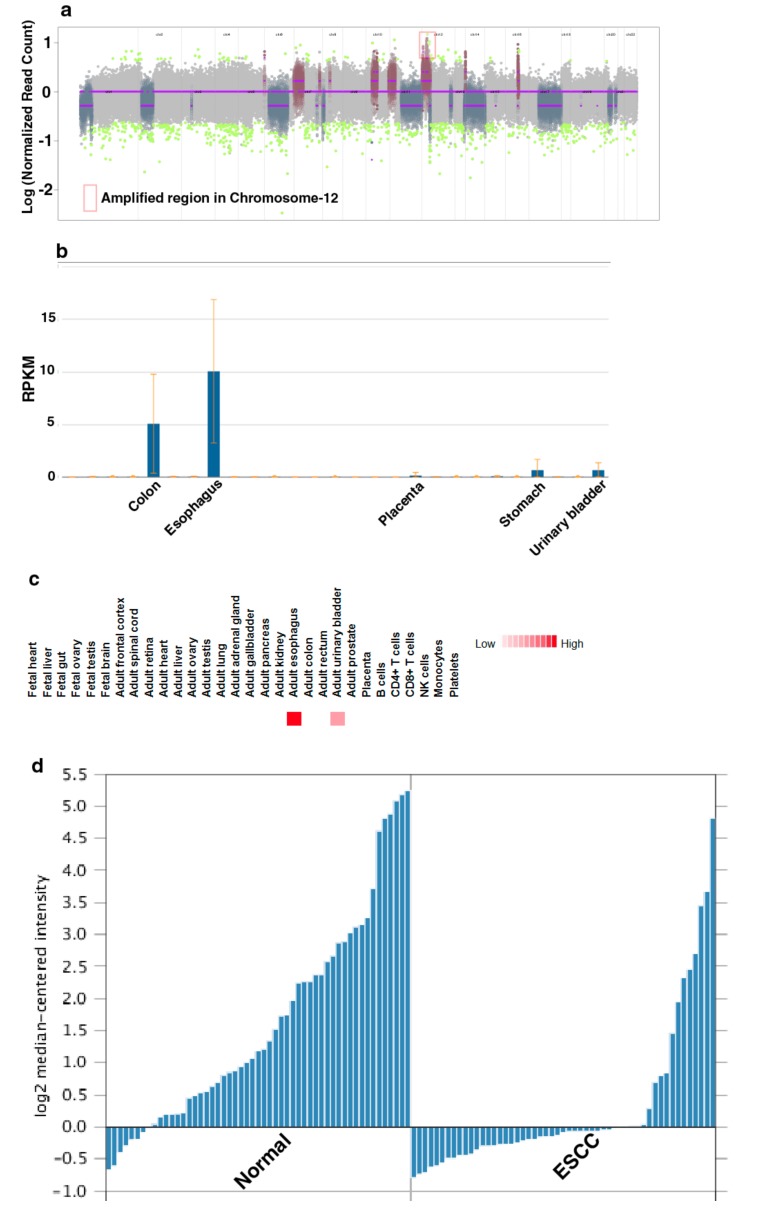
Keratin 24 (KRT24) shows enriched expression in esophagus and is downregulated in esophageal squamous cell carcinoma (ESCC). (**a**) Genome-wide copy number changes in Het1A-8M cells. (**b**) Relative expression of KRT24 mRNA across various human tissues. Adapted from NCBI Gene page for KRT24 (https://www.ncbi.nlm.nih.gov/gene/192666) (**c**) Relative expression of KRT24 protein across various human tissues. Adapted from http://www.humanproteomemap.org (**d**) Relative expression of KRT24 in esophageal squamous cell carcinoma and adjacent normal mucosa [35].

To characterize genomic alterations associated with chronic tobacco exposure, we carried out exome sequencing of Het1A-P and Het1A-8M cells (Supplementary Materials Figure S1). For each cell line, we achieved a target depth of ~100× with 99% target coverage. Using OncoCNV, we identified amplifications in 110 genes (copy number ≥ 3, *p* < 0.001) in tobacco-treated cell line Het1A-8M (Supplementary Materials Table S1). A large majority of these genes are known to be amplified in various cancers. Notably, 70 of the 110 genes were found to be located on chromosome 12 (Figure 2a). Chromosome 12 is known to harbor numerous genes associated with cancer [24]. Amplification of genes on chromosome 12 is previously reported in ESCC [30,31]. The amplified genes were found to be involved in transmembrane signaling receptor activity, molecular transducer activity and G-protein coupled receptor activity.

A total of 254 SNVs corresponding to 203 genes were identified in Het1A-8M cells compared to parental cells (Supplementary Materials Table S2). Of these, 78 were non-synonymous mutations. While 72 were missense mutations, six were found to be stop-gain mutations. 30 genes where missense mutations were identified in the current study are previously reported to be mutated in ESCC [32,33]. This included E74 like ETS transcription factor 3 (ELF3), spen family transcriptional repressor (SPEN) and notch receptor 3 (NOTCH3) that are known to be mutated in various cancers. Interestingly, we observed a nonsense mutation in keratin 24 (KRT24) that resulted in premature stop codon. KRT24 is an eight exon gene that encodes a 525 amino acid protein. Premature stop codon occurs in the first exon that would potentially result in degradation of this transcript by nonsense-mediated decay. KRT24 shows tissue-specific expression pattern in colon and esophagus [34] (Figure 2b,c). However, the role of KRT24 in these tissues is poorly characterized. Previous gene expression studies [35,36] have shown significant downregulation of KRT24 expression in ESCC compared to adjacent normal tissues (Figure 2d). A recent study has suggested KRT24 as a potential anti-proliferative factor [37]. Considering abundant expression of KRT24 in normal esophagus and loss of expression in ESCC, we speculate a tumor suppressor role for KRT24 in ESCC.

### 3.3. Global Proteomic Profiling Shows Differentially Expressed Proteins Associated with Chronic Exposure to Chewing Tobacco

**Figure 3:**
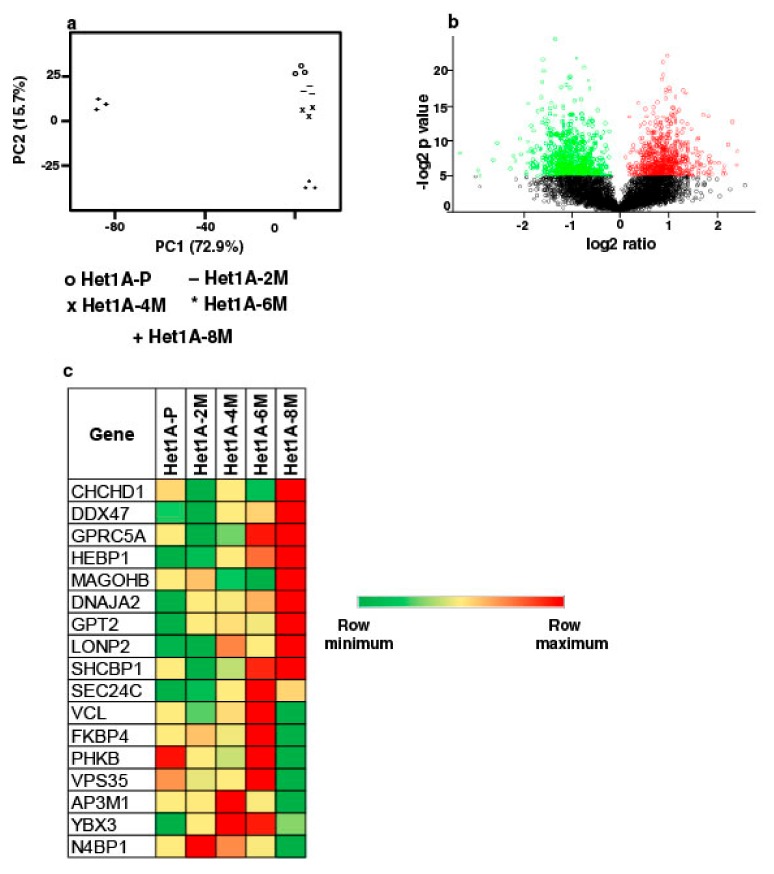
Proteomic analysis of chewing tobacco treated esophageal epithelial cells. (**a**) Principal component analysis of parental and chewing tobacco exposed Het1A cells. (**b**) Volcano plot showing differential expression of proteome in Het1A-8M cells compared to parental Het1A cells. (**c**) Heat map showing expression pattern of a subset of proteins encoded by genes that showed amplification in Het1A-8M cells.

To elucidate proteome-wide changes associated with chewing tobacco exposure, we employed TMT-based quantitative proteomic profiling strategy. We compared global protein expression pattern across Het1A-P, Het1A-2M, Het1A-4M, Het1A-6M and Het1A-8M cells (Supplementary Materials Figure S2). A total of 4446 proteins were identified and quantified across three replicates. A complete list of all proteins identified in the current study is provided in Supplementary Materials Table S3. Proteins that showed ≥1.5 fold difference (*p* ≤ 0.05) in expression compared to Het1A-P were considered differentially expressed. We found 22, 17, 40 and 462 proteins to be overexpressed, whereas 9, 4, 14 and 434 proteins were downregulated in Het1A-2M, Het1A-4M, Het1A-6M and Het1A-8M, respectively. Phenotypic assays had shown significantly increased proliferation, invasion and colony formation ability in Het1A-8M cells. Similarly, we observed the largest number of differentially expressed proteins in Het1A-8M cells. Principal component analysis (PCA) revealed distinct clustering of Het1A-8M compared to earlier time points suggesting distinct molecular profile associated with this time point (Figure 3a). As most changes were observed in Het1A-8M cells, all the analysis was carried out using this dataset. A volcano plot showing differentially expressed proteins in Het1A-8M is shown in Figure 3b.

We integrated proteomic data with CNVs identified from exome dataset of same cells. Of the 110 genes that were identified as amplified in exome dataset, 17 were identified in proteomic dataset in tobacco treated cells. While 9 of these 17 were overexpressed in Het1A-8M, the others were overexpressed in Het1A-6M, an earlier time point (Figure 3c). This indicates that amplifications had potential functional consequences.

A fine balance exists between proliferative and anti-proliferative signals in a cell. This balance is dysregulated during cell transformation. Epidermal growth factor receptor (EGFR), a receptor tyrosine kinase and a well-known oncogene, was found to be overexpressed in Het1A-8M (1.7 fold). Tumor suppressor genes of the DNA mismatch repair MutS family were found to be downregulated (MSH2—0.5 fold, MSH6—0.4 fold). PP2A is a serine/threonine phosphatase that regulates signaling events and considered as an “off switch” in cancer signaling [38]. Both the catalytic and regulatory subunits of PP2A were found to be downregulated in Het1A-8M (PPP2CA—0.5 fold, PPP2R1A—0.5 fold, PPP2R1B—0.5 fold, PPP2R4—0.4 fold and PPP2R5D—0.6 fold).

Carcinogens in tobacco cause an increase in ROS within cells. A number of enzymes play a role in ROS scavenging. This scavenging system helps the cells to adapt to high ROS condition and thrive [39]. Proteomic data showed that enzymes involved in ROS scavenging such as superoxide dismutase (SOD2—2.7 fold), glutathione peroxidise (GPX8—2.1 fold) and peroxiredoxins (PRDX3—1.7 fold, PRDX4—1.8 fold) were overexpressed in Het1A-8M.

### 3.4. Protein Expression Pattern of Het1A-8M Shows Elevated Expression of Cancer Stem Cell (CSC) Markers

CSCs are known to possess the property of self-renewal along with capabilities of tumor initiation and progression. Cancer stem cell subpopulations have been characterized based on expression of distinct surface markers [40]. Studies have shown that CD44 [41] and ICAM1 [42] are cancer stem cell markers in ESCC. We observed that both CD44 (2.6 fold) and ICAM1 (3.7 fold) were overexpressed in Het1A-8M cells. Therefore, we probed the proteomic data for expression pattern of other well-known cancer stem cell markers such as ALDH1A1 (2.6 fold), CD151 (2.1 fold), CD47 (1.9 fold) and THY1 (3.5 fold). In addition, we compared proteomic data with two earlier studies [42,43] where differential proteomic analysis was carried out comparing ESCC cancer stem cells and non-stem cells (Table 1). Interestingly, Het1A-8M protein expression data showed elevated expression of several ESCC cancer stem cell markers that have been reported before. 

### 3.5. Esophageal Cells Chronically Exposed to Tobacco Extract Show Metabolic Reprogramming and Higher Mitochondrial Mass

**Figure 4:**
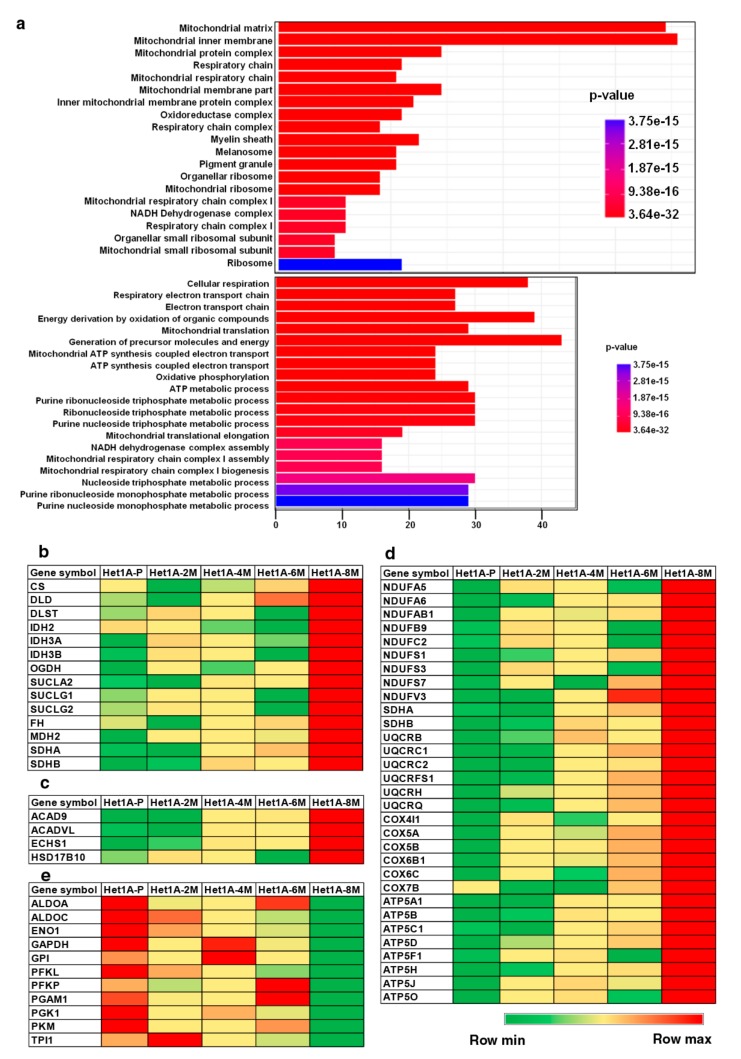
Chronic exposure to chewing tobacco leads to metabolic reprogramming. (**a**) Gene ontology (GO) enrichment of cellular component and biological process associated with overexpressed proteins in Het1A-8M cells. Heat maps showing relative expression of proteins involved in (**b**) tricarboxylic acid (TCA) Cycle, (**c**) fatty acid oxidation, (**d**) oxidative phosphorylation (OXPHOS) and (**e**) glycolysis.

Gene set enrichment analysis of overexpressed proteins in Het1A-8M showed significant enrichment of Gene Ontology (GO) term “mitochondria”, in the GO categories cellular component and biological process (Figure 4a). The human mitochondrial proteome consists of 1158 proteins [44]. While 13 of these are encoded by mitochondrial DNA (mtDNA), the others are encoded by nuclear DNA and transported to mitochondria. In the current study, we identified 711 proteins that are known to be localized in the mitochondria, out of which 229 were found to be differentially expressed.

Mitochondria are known as the “powerhouse of the cell”. It is an important organelle that is highly metabolically active due to numerous biochemical pathways that occur in them [45]. Fatty acid oxidation, tricarboxylic acid (TCA) cycle and oxidative phosphorylation (OXPHOS) are the major energy pathways that occur in the mitochondria.

Metabolic reprogramming is a hallmark of cancer [9]. Since the last few decades, aerobic glycolysis or the “Warburg effect” was the most accepted form of metabolic reprogramming. This theory states that cancer cells primarily rely on glycolysis for their ATP requirement. Pyruvate, the end product of glycolysis, is converted into lactate and used for various anabolic processes in the cell [12]. However, many recent studies have demonstrated that a subset of cancer cells, especially cancer stem cells, utilize OXPHOS for their energy requirements and do not rely on glycolysis [46,47]. Increased mitochondrial biogenesis and high mitochondrial mass is considered as a biomarker for CSCs [48,49]. We found that proteins involved in energy pathways including TCA cycle, OXPHOS and fatty acid oxidation were overexpressed in Het1A-8M (Figure 4b–d). On the contrary, enzymes involved in glycolytic pathway were downregulated (Figure 4e). These data indicate that tobacco treated cells have undergone a metabolic switch and also provided further evidence that Het1A-8M cells resembled CSCs. 

**Figure 5:**
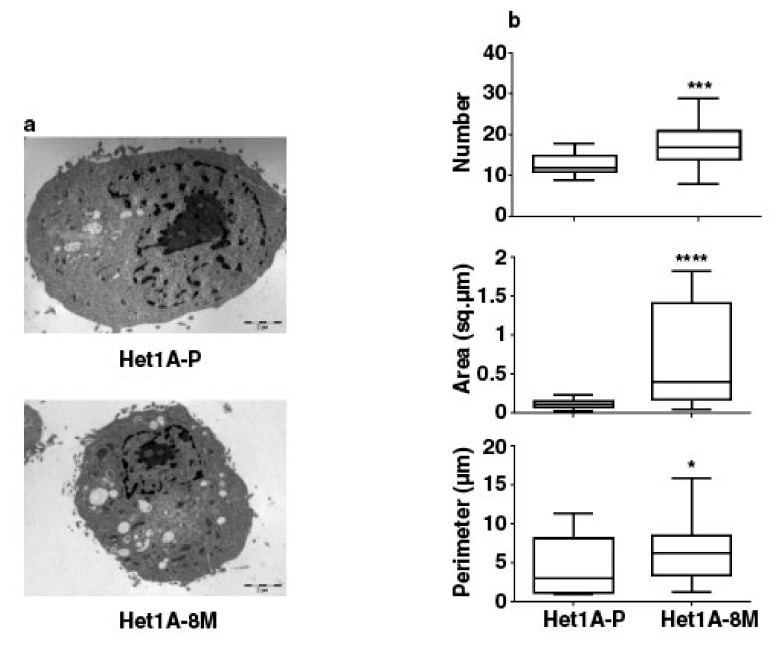
Chronic exposure to chewing tobacco increases mitochondrial mass in Het1A cells. (**a**) Electron micrographs of parental and Het1A-8M cells. (**b**) Relative quantitation of number, size and perimeter of mitochondria in parental and smokeless tobacco exposed Het1A cells. A total of 50 cells were analyzed. * (*p* ≤ 0.05), *** (*p* ≤ 0.001), **** (*p* ≤ 0.0001).

We performed electron microscopy to investigate changes in mitochondrial number and size in Het1A-8M cells (Figure 5a). We saw that the number, area and perimeter of mitochondria were significantly higher in the chewing tobacco treated cells (Figure 5b). Het1A-8M cells showed higher mitochondrial mass, similar to CSCs. In summary, the expression pattern of CSC markers, metabolic enzymes and increased mitochondrial mass evident by electron microscopy demonstrates that chronic exposure to chewing tobacco leads to a CSC-like phenotype.

## 4. Discussion

Tobacco is a well-known risk factor for several cancers. Global consumption of tobacco is predominantly in the form of cigarette smoke. However, in several countries in South Asia including India, tobacco is consumed in its smokeless form by chewing the tobacco. Smokeless/chewing form of tobacco is a well-known risk factor of ESCC. However, molecular alterations associated with exposure to chewing tobacco are not well characterized. We developed an in vitro model of chronic exposure to chewing tobacco using a non-neoplastic esophageal epithelial cell line. Chronic treatment of these cells resulted in cellular transformation after eight months of treatment as confirmed by phenotypic assays. These cells were molecularly characterized by carrying out whole exome sequencing and global proteomic profiling to determine molecular alterations associated with transformed cell phenotype. Exome sequencing revealed several single nucleotide variations and amplifications following chewing tobacco treatment.

Proteomic profiling provided significant insights into molecular mechanisms associated with oncogenic phenotype in chewing tobacco treated cells. The cells had acquired an OXPHOS phenotype. We observed a significant increase in size and number of mitochondria in chewing tobacco exposed cells. In addition, we observed elevated expression of numerous mitochondrial proteins. OXPHOS phenotype has been reported in a number of cancer types. It is shown to be a characteristic feature of cancer stem cells. Cancer cells that show OXPHOS phenotype are vulnerable for complex I inhibitors such as metformin [50] and antibiotics [51]. Considering that CSCs are often resistant to conventional therapy, these alternative strategies that exploit the metabolic differences in CSCs may prove efficacious. These observations warrant further investigation in primary tumors from ESCC patients with a history of chewing tobacco.

## Figures and Tables

**Table 1:** List of proteins that are reported to be overexpressed in ESCC cancer stem cells and found to be overexpressed in Het1A-8M. (+ implies overexpressed. Previous studies do not report a fold change value).

Gene Symbol	Tsai et al.	Huang et al.	Het1A-8M/Het1A-P (Relative Fold Change)
HLA-B	+	Not reported	2.9
ICAM1	+	Not reported	3.7
HLA-A	+	Not reported	3.1
RAB9A	+	Not reported	2.2
SLC3A2	+	Not reported	2.2
SLC25A1	+	Not reported	1.7
MMGT1	+	Not reported	1.6
PI4K2A	+	Not reported	1.7
CYP1B1	Not reported	+	2.8
CLK1	Not reported	+	2.2
LEMD3	Not reported	+	1.8
DDX10	Not reported	+	1.7
RBM15	Not reported	+	1.4
RANBP2	Not reported	+	1.7
KIF14	Not reported	+	1.8
CDCA2	Not reported	+	1.5

## Supplementary Materials

The following are available online at https://www.mdpi.com/2073-4409/8/9/949/s1: Figure S1—Methodology followed for exome data acquisition and analysis. Figure S2—Methodology followed for proteomic data acquisition and analysis. Figure S3—Quantification of western blot images in Figure 1f. * (*p* ≤ 0.05), ** (*p* ≤ 0.01), *** (*p* ≤ 0.001), **** (*p* ≤ 0.0001), ns (non-significant). Table S1—List of all amplifications identified in Het1A-8M (copy number ≥ 3, *p* < 0.001). Table S2—List of all SNVs identified in Het1A-8M. Table S3—List of all proteins identified in the current study.
