# Supplementary material for: Chronic Exposure to Chewing Tobacco Induces Metabolic Reprogramming and Cancer Stem Cell-Like Properties in Esophageal Epithelial Cells
Source: Cells. 2019 Aug 21;8(9):949. doi: 10.3390/cells8090949 (PMC6770059; doi:10.3390/cells8090949)

Supplementary Figure S1

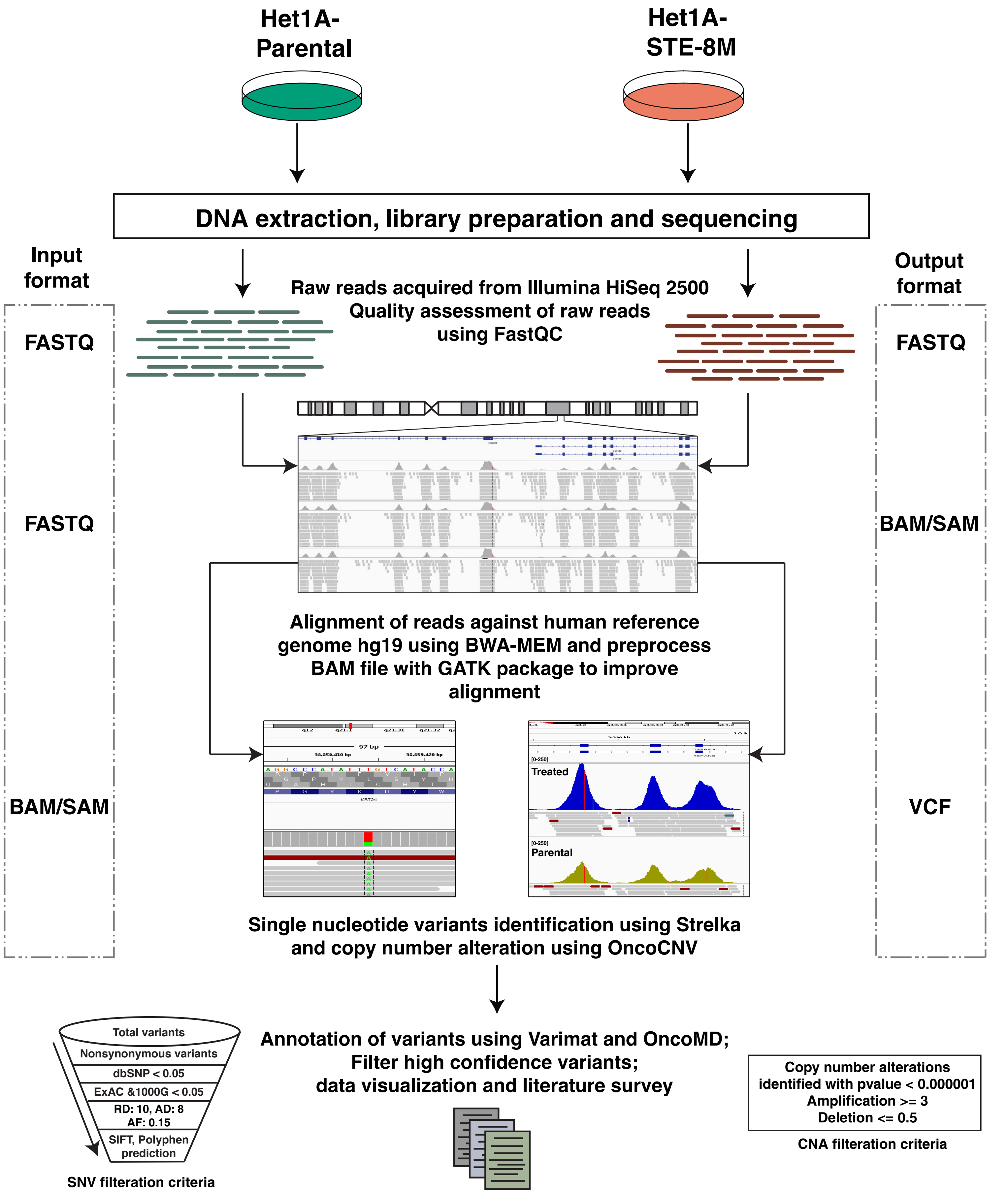

Supplement: Supplementary file 1 [file cells-08-00949-s001.zip › cells-550609-supplementary/Het1A-Tobacco_all files/Supp.Fig.S1.pdf]
